# Chemical Composition of Four Essential Oils of *Eugenia* from the Brazilian Amazon and Their Cytotoxic and Antioxidant Activity

**DOI:** 10.3390/medicines4030051

**Published:** 2017-07-08

**Authors:** Joyce Kelly R. da Silva, Eloisa Helena A. Andrade, Leilane H. Barreto, Nádia Carolina F. da Silva, Alcy F. Ribeiro, Raquel C. Montenegro, José Guilherme S. Maia

**Affiliations:** 1 Programa de Pós-Graduação em Biotecnologia, Universidade Federal do Pará, Belém 66075-110, Brazil; joycekellys@ufpa.br (J.K.R.d.S.); rcm.montenegro@gmail.com (R.C.M.);; 2 Programa de Pós-Graduação em Química, Universidade Federal do Pará, Belém 66075-110, Brazil; eloisandrade@ufpa.br (E.H.A.A.); favacho@ufpa.br (A.F.R.); 3 Coordenação de Botânica, Museu Paraense Emilio Goeldi, Belém 66077-530, Brazil; 4 Laboratório de Citogenética Humana, Universidade Federal do Pará, Belém 66075-900, Brazil; leilanebarreto@yahoo.com.br (L.H.B.); nadia_fonseca_silva@hotmail.com (N.C.F.d.S.); 5 Programa de Pós-Graduação em Recursos Naturais da Amazônia, Universidade Federal do Oeste do Pará, Santarém 68035-110, Brazil

**Keywords:** *Eugenia egensis*, *E. flavescens*, *E. patrisii*, *E. polystachya*, Myrtaceae, essential oil, cytotoxicity, antioxidant activity

## Abstract

**Background:** *Eugenia* species are appreciated for their edible fruits and are known as having anticonvulsant, antimicrobial and insecticidal actions. **Methods:** The plant material was collected in the southeastern Pará state of Brazil and submitted to hydrodistillation. GC-MS analyzed the oils, and their antioxidant and cytotoxic activities were evaluated by the DPPH and MTT assays. **Results:** The main components identified in the *Eugenia* oils were 5-hydroxy-*cis*-calemene, (2*E*,6*E*)-farnesol, (2*E*,6*Z*)-farnesol, caryophylla-4(12),8(13)-dien-5α-ol-5β-ol, *E*-γ-bisabolene, β-bisabolene, germacrene D, and ishwarane. The oil of *E. egensis* showed the most significant antioxidant activity (216.5 ± 11.6 mg TE/mL), followed by the oils of *E. flavescens* (122.6 ± 6.8 mg TE/mL) and *E. patrisii* (111.2 ± 12.4 mg TE/mL). *Eugenia* oils were cytotoxic to HCT-116 (colon cancer) cells by the MTT assay, where the most active was the oil of *E. polystachya* (10.3 µg/mL), followed by the oils of *E. flavescens* (13.9 µg/mL) and *E. patrisii* (16.4 µg/mL). The oils of *E. flavescens* and *E. patrisii* showed the highest toxicity for MRC5 (human fibroblast) cells, with values of 14.0 µg/mL and 18.1 µg/mL, respectively. **Conclusions:** These results suggest that *Eugenia* oils could be tested in future studies for the treatment of colon cancer and oxidative stress management.

## 1. Introduction

Myrtaceae Juss. comprises 142 genera and about 5500 species of trees and shrubs, distributed in the tropical and subtropical regions of the world, with centers of diversity in Tropical America and Oceania, and a few species in Africa [1]. In Brazil, it is one of the most diverse and is mainly represented by fruit trees. Twenty-three genera and about 1000 species were found and all belonged to the Myrtoideae subfamily and Myrteae tribe [2,3]. *Eugenia* L. is one of the largest genera within Myrtaceae and 388 species are native in Brazil [3]. *Eugenia egensis* DC., common name “cambuí” (syn. *E. egensis* var. *grandifolia* O. Berg, *E. erythrocarpa* Barb. Rodr., *E. parodiana* Morong, *E. perforata* O. Berg, *E. pothaplosantha* Barb. Rodr., *E. sphaerosperma* DC., *E. tenuiramis* Miq.), *Eugenia flavescens* DC., common name araçá-da-mata” (syn. *E. flavescens* var. *guianensis* Sagot), *Eugenia patrisii* Vahl, known as “ubaia-rubí” (syn. *E. berlynensis* O. Berg, *E. inocarpa* DC., *E. parkeriana* DC., *E. tefeensis* O. Berg, *E. vellozii* O. Berg., *stenocalyx patrisii* (Vahl) O. Berg), and *Eugenia polystachya* Rich. (syn. *E. forsteri* O. Berg, *E. schlechtendaliana* O. Berg) [4] are shrubs or small trees of 2–5 m, with a wide occurrence in the Brazilian Amazon.

The Myrtaceae family is known for the high terpene concentration of the foliage and the considerable qualitative and quantitative variation in foliar terpenes at taxonomic, population, and individual levels [5,6]. Many *Eugenia* species are appreciated for their edible fruits, such as *E. uniflora* L. (pitanga), *E. involucrata* DC. (cereja-do-mato), *E. jambolana* Lam. (jamelão), *E. pyriformis* Cambess. (uvaia), and *E. dysenterica* DC. (cagaita). Beyond the volatiles of the fruits, these species also accumulate essential oils in their leaves [7,8,9,10,11]. Sesquiterpene hydrocarbons and oxygenated sesquiterpenes predominate in the essential oils of *Eugenia* and they are from the germacrane, caryophylane, and guaiane types [12,13].

Essential oils of *Eugenia* have significant biological activities, such as anticonvulsant [14], antibacterial [15], antifungal [16], antiparasitic [17], and insecticidal [18] activities. Additionally, some *Eugenia* oils have been reported as antioxidant and cytotoxic. The oil of *Eugenia caryophyllata* (clove) (syn. *Syzygium aromaticum* (L.) Merril & Perry) is rich in eugenol and is a powerful natural antioxidant, with different mechanisms of action, such as radical scavenging, metals chelation, and the inhibition of lipid peroxidation [19]. Furthermore, clove oil showed cytotoxic activity and the induction of apoptosis in human promyelocytic leukemia cells (HL-60) [20].

The aim of the present study was to analyze the composition of the oils of *Eugenia egensis*, *E. flavescens*, *E. patrisii*, and *E. polystachya*, and evaluate their cytotoxic and antioxidant properties.

## 2. Materials and Methods

### 2.1. Plant Material

Botanical material (aerial parts, 500 g each plant) was collected in three municipalities located in the Southeast Pará state, Brazil, during the rainy season (January 2011). *Eugenia egensis* DC. (MG 181220) was collected in the city of Marabá. *Eugenia flavescens* DC. (MG 200127) and *E. polystachya* Rich. (MG191868) were sampled in the Carajás National Forest, in the town of Parauapebas. *Eugenia patrisii* Vahl. (MG 200132) was collected in the city of São Geraldo do Araguaia. *Eugenia* species vouchers were deposited in the Herbarium of Emilio Goeldi Museum (MG), the city of Belém, Pará state, Brazil.

### 2.2. Plant Processing and Extraction of the Essentials Oils

Aerial parts (leaves and thin stems) of the plants were air-dried, grinded, and submitted to hydrodistillation using Clevenger-type apparatus (100 g, 3 h). The oils were dried over anhydrous sodium sulfate, and their percentage contents were calculated by the plant dry weight. The moisture contents of the samples were computed after phase separation using a Dean–Stark trap (5 g, 60 min) and toluene as the solvent phase.

### 2.3. Oil Composition Analysis

Analyses of the oils were carried out on a GC-MS Thermo-Electron model Focus DSQ II (Thermo Fisher Scientific, Waltham, MA, USA), under the following conditions: DB-5ms (30 m × 0.25 mm; 0.25 mm film thickness) fused-silica capillary column (Agilent J&W GC Columns, Santa Clara, CA, USA); programmed temperature, 60–240 °C (3 °C/min); injector temperature, 250 °C; carrier gas, helium, adjusted to a linear velocity of 32 cm/s (measured at 100 °C); injection type, split (1.0 μL), from 1:1000 hexane solution; split flow was adjusted to yield a 20:1 ratio; septum sweep was a constant 10 mL/min; EIMS, electron energy, 70 eV; temperature of the ion source and connection parts, 200 °C. The quantitative data regarding the volatile constituents were obtained by peak area normalization using a FOCUS GC/FID (Thermo Fisher Scientific, Waltham, MA, USA) operated under similar conditions for the GC–MS, except the carrier gas, which was nitrogen. The retention index was calculated for all the volatile constituents using a homologous series of *n*-alkanes (C_8_-C_32_, Sigma–Aldrich, St. Louis, MO, USA), according to Van den Dool and Kratz (1963) [21].

### 2.4. Antioxidant Assay

The antioxidant activity of the *Eugenia* oils was determined by the DPPH radical scavenging assay. DPPH is a stable dark violet free radical with a maximum absorption at 517 nm, which is reduced in the presence of antioxidants. Each sample (5 µL) was mixed with Tris-HCl buffer (100 mM, 900 µL, pH 7.4), ethanol (40 µL), and Tween 20 solution (0.5%, 50 µL, *w*/*w*), and was then added to DPPH (0.5 mM, 1 mL) in ethanol. The standard curves were prepared using Trolox and BHA (1.0 to 8.0 μg/mL), which are standards of hydrosoluble and liposoluble antioxidants, respectively. The results were expressed as milligrams of Trolox (mgTE/mL) and BHA (mg BHAE/mL) equivalents per milliliter of the sample [22].

### 2.5. Cytotoxicity Assay (Against Cancer Cell Lines)

The MTT colorimetric assay was used to measure the cell metabolic activity [23]. The oils (0.2 to 25 μg/mL) were tested for cytotoxic activity against three cancer cell lines: HCT-116 (colon), SKMEL19 (melanoma), AGP-01 (gastric). All cell lines were maintained in DMEM (Dulbecco’s Modified Eagle Medium) medium supplemented with fetal bovine serum (10%), glutamine (2 mM), penicillin (100 U/mL), streptomycin (100 µg/mL) at 37 °C with 5% CO_2_. Each oil was dissolved in DMSO to obtain a concentration of 10 mg/mL. The final concentration of DMSO in the culture medium was kept constant, below 0.1% (*v*/*v*). Essential oils (25 μg) were incubated with the cells for 72 h. The negative control received the same amount of DMSO (0.001% in the highest concentration). The cell viability was determined by reduction of the yellow dye 3-(4,5-dimethyl-2-thiazol)-2,5-diphenyl-2*H*-tetrazolium bromide (MTT) to a blue formazan product. Doxorubicin and eugenol were the positive controls.

### 2.6. Cell Membrane Disruption

The potential of the cell membrane lyses was evaluated by the release of the hemoglobin in the medium. The test was performed in 96-well plates using mouse hemoglobin suspension (2%) in NaCl solution (0.85%), containing CaCl_2_ (10 mM). The oils, diluted as mentioned above, were tested at 200 μg/mL. After incubation at room temperature for 1 h, followed by centrifugation, the supernatant was removed, and the liberated hemoglobin was measured spectrophotometrically at 540 nm. DMSO was used as the negative control and Triton X-100 (1%) was employed as the positive control [24].

### 2.7. Statistical Analysis

Samples were assayed in triplicate, and the results are shown as means ± standard deviation. Analysis of variance was conducted, and the differences between variables were tested for significance by a Tukey test. Differences at *p* < 0.05 were considered statistically significant. The *IC*_50_’s values were calculated by nonlinear regression using the GraphPad program (version 5.0, Intuitive Software for Science, San Diego, CA, USA).

## 3. Results and Discussion

### 3.1. Essential Oil Composition

The dried leaves and fine stems (aerial parts) of *Eugenia egensis*, *E. flavescens*, *E. patrisii*, and *E. polystachya* provided oil yields of 2.5%, 1.0%, 0.7%, and 1.0%, respectively. Individual components were identified by comparison of both mass spectrum and GC retention data with authentic compounds, previously analyzed and stored in the data system. Furthermore, they were identified with the aid of commercial libraries containing the retention indices and mass spectra of volatile compounds, commonly found in essential oils [25,26]. One hundred volatile compounds were identified, corresponding to an average of 93% of the total composition of the oils (Table 1). Sesquiterpenes were the most highly represented class, as many hydrocarbons and oxygenated constituents.

The principal constituents (above 5%) of *E. egensis* oil were 5-hydroxy-*cis*-calemenene (35.8%), β-caryophyllene (8.9%), *trans*-cadina-1,4-diene (6.3%), *trans*-calamenene (6.1%), *trans*-muurola-3,5-diene (5.9%), and ledol (5.0%). These sesquiterpenes were grouped in accordance with the following biosynthetical pathways: cadinane, muurolane, and caryophyllane. *Eugenia polystachya* oil was dominated by germacrene D (18.4%), ishwarane (15.7%), 7-*epi*-α-selinene (7.5%), and bicyclogermacrene (5.1%). Therefore, sesquiterpenes presented the germacrane and selinane structural-types. To our knowledge, it is the first study on the composition of the *E. egensis* and *E. polystachya* oils, and the first report on the occurrence of 5-hydroxy-cis-calemenene as a significant constituent in *Eugenia* essential oils. Other sesquiterpenes with a germacrane skeleton have been reported in *E. protenta* [27] and *E. uniflora* [28].

*Eugenia flavescens* oil is rich in (*E*)-γ-bisabolene (35.0%), β-bisabolene (34.7%), and (*E*)-iso-γ-bisabolene (5.1%), comprising about 75.0% of the total oil composition. A previous study reported the occurrence of germacrene D and bicyclogermacrene in the oil of a specimen of *E. flavescens* collected in the city of Maracanã, Pará State, Brazil [29]. A notable occurrence of the bisabolane skeleton was described in other Myrtaceae species. α-Bisabolene occurs in the oils of *Myrcia splendens* [13], *M. fallax* and *M. glabra* [30], *M. obtecta* [31], *M. laruotteana* [32], and *M. bracteata* [33]. The oxygenated sesquiterpenes (2*E*,6*E*)-farnesol (34.5%), (2*E*,6*Z*)-farnesol (23.2%), and the mixture of caryophylla-4(12),8(13)-dien-5α-ol and caryophylla-4(12),8(13)-dien-5β-ol (15.6%) were the main constituents of the oil of *Eugenia patrissii*. Therefore, sesquiterpene compounds belong to the caryophyllane and acyclic groups. Another sample of *E. flavescens*, collected in the city of Maracanã, Pará State, Brazil, different to the sample studied in this paper, showed the hydrocarbon sesquiterpenes trans-cadina-1,4-diene, trans-muurola-3,5-diene, and β-caryophyllene, as the primary components [29].

The difference found in the main constituents of the oils of *E. flavescens* and *E. patrissii*, when comparing them separately with the previously described oils [29], is due to the presence of two distinct chemotypes, whose specimens were sampled at different collection sites; the first in a secondary forest area in the northeast of Pará and the second in an area of savanna in the south of Pará, Brazil, with a very diverse soil and climate environment, and a distance between them of about 1000 km.

### 3.2. Antioxidant Activity

**Figure 1:**
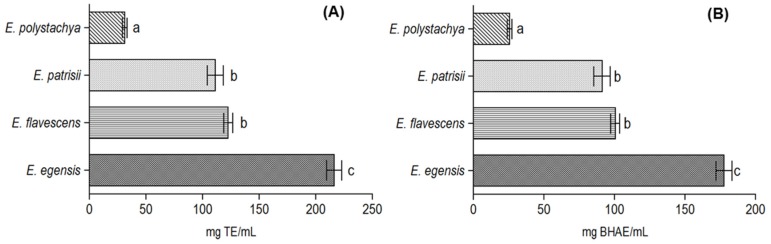
Antioxidant activity of the *Eugenia* oils by the DPPH assay. Results expressed in milligrams of Trolox (**A**) and BHA (**B**) equivalent per milliliter of sample. Mean ± standard deviation (*n* = 3). Values with different letters in the figure represents statistical differences at the *p* < 0.05 level (Tukey’s test).

The radical scavenging activity using the DPPH radical (2,2-diphenyl-1-picrylhydrazyl) was tested with the different *Eugenia* oils, and the absorbance at 517 nm was observed. Regarding percentage values, the inhibiting activity (over 120 min) was calculated in the following order: *E. egensis* (79.6 ± 4.3%), *E. flavescens* (45.1 ± 2.5%), *E. patrisii* (40.9 ± 4.6%), and *E. polystachya* (11.5 ± 1.3 %). The antioxidant activity was expressed in comparison with the Trolox and BHA standards (Figure 1).

The highest activity observed for the *E. egensis* oil (TEAC = 216.5 ± 11.6 mg TE/mL and 177.6 ± 9.8 mg BHAE/mL) could be attributed to the oxygenated sesquiterpene 5-hydroxy-*cis*-calamenene. The presence of a phenolic ring in the structure of 5-hydroxy-*cis*-calamenene enhances the antioxidant activity due to its ability for scavenging free radicals, the donation of hydrogen atoms or electrons, or chelation with metal cations [34]. The oil of *Croton cajucara* Benth. that contains 33.0% of the isomer 7-hydroxy-calamenene, showed significant antioxidant activity in the DDPH assay and its *IC*_50_ value was 35.6 µg/mL, only four times less active than rutin (*IC*_50_ 9.3 µg/mL) [35]. Additionally, it was reported that the isomer 8-hydroxy-calamenene could attenuate the retinal damage significantly, a common risk factor for glaucoma disease caused by oxidative stress, exhibiting neuroprotective effects when tested in vitro and in vivo [36].

*Eugenia flavescens* (122.6 ± 6.8 mg TE/mL and 100.6 ± 5.4 mg BHAE/mL) and *E. patrisii* (111.2 ± 12.4 mg TE/mL and 91.3 ± 10.1 m BHAE/mL) oils showed moderate antioxidant activity. The main constituents of these two oils are sesquiterpene hydrocarbons with bisabolane and farnesane type structures, respectively (see Table 1). The oil of *Psammogeton canescens* Vatke, rich in β-bisabolene (33.4%), showing significant antioxidant activity in the DPPH assay, was previously reported and strengthens our results [37]. On the other hand, to the present date, no information has been reported on the antioxidant activity of the farnesol isomers.

### 3.3. Cytotoxic Activity

Among many valuable plant products, the essential oils are used in complementary medical treatment strategies. The action of essential oils and their constituents has been studied for a variety of cancer types [38]. In the present study, the antiproliferative effect of different *Eugenia* oils was tested against three human cancer cell lines (AGP-01, HCT-116, and SKMEL-19) and one normal human fibroblast cell line (MRC-5), using the MTT assay. The *IC*_50_ values were determined after 72 h exposure, as shown in Table 2. *Eugenia flavescens*, *E. patrisii*, and *E. polystachya* oils presented cytotoxicity against the HCT-116 colon cancer cell line, except for the oil of *E. egensis*, which did not display cytotoxicity against any of the cells until the concentration of 25 µg/mL. The oil of *E. polystachya* was the most active, with an *IC*_50_ value of 10.3 µg/mL. Germacrene D was the main constituent of this oil (18.4%) and, previously, it was identified with a significant percentage in the oils of *Guatteria diospyroides*, *G. oliviformis*, and *Unonopsis costaricensis*. These Annonaceae oils showed remarkable cytotoxic activities against MDA-MB-231 cells (human breast tumor) [39]. The germacrane group was also highlighted in the study of the essential oil of *Porcelia macrocarpa*, another Annonaceae species. A mixture of germacrene D and bicyclogermacrene isolated from its oil showed significant cytotoxic potential against HL-60 cells (human leukemia) [40]. As mentioned before, the oil of *Eugenia caryophyllata* (clove) exhibits strong cytotoxic activity in HL-60 cells [20], as well as being active against colon and melanoma cancer cells [41,42]. Thus, the results in Table 2 were compared to eugenol, the major constituent of clove oil. Interestingly, the oil of *E. polystachya* exhibits no activity against the normal MRC-5 fibroblast cell line, whereas the other two oils from *E. flavescens* and *E. patrisii* display the same range of cytotoxicity on HCT-116 cancer cells and in MCR-5 normal cells, what makes them suitable for further investigation. To our knowledge, this is the first time that the cytotoxic activity of these oils has been reported. All tested oils did not display lytic activity against red blood cells (see Table 2).

Antioxidants are believed to be directly antimutagenic [43] and anticarcinogenic due to their radical scavenging properties [44,45]. The oil of *E. egensis* showed significant antioxidant activity, but no cytotoxicity against cancer cell lines (*IC*_50_ > 25.0 µg/mL). These results should be attributed to the presence of the phenolic ring in the structure of 5-hydroxy-*cis*-calamenene, as many phenolic compounds are reported as cytoprotectives [46].

## 4. Conclusions

Our investigation of the chemical profile of the *Eugenia* essential oils has contributed to chemosystematic studies of Myrtaceae species, for which the occurrence of bisabolane-type skeletons and acyclic sesquiterpenes as important characteristics have been reported. Additionally, it is the first report for the presence of 5-hydroxy-*cis*-calamenene in *Eugenia* oils. The essential oils showed significant antioxidant activity and selective cytotoxicity against HCT-116 cancer cells (colon) and did not promote membrane damage. The results suggest that *Eugenia* oils could be tested in future studies for the treatment of colon cancer and oxidative stress management.

## Figures and Tables

**Table 1:** Composition (%) of the oils of *Eugenia* species.

Constituents	RI_Calc._	RI_Lit._	*Eugenia egensis*	*Eugenia flavescens*	*Eugenia patrisii*	*Eugenia polystachya*
Limonene	1026	1024				0.1
α-Terpineol	1187	1186			0.1	
Thymol	1290	1289			0.1	
δ-Elemene	1337	1335	1.6	0.5	0.3	4.1
α-Cubebene	1348	1345	2.2			0.1
α-Ylangene	1374	1373	0.1			0.1
α-Copaene	1376	1374	1.6		0.1	0.6
β-Bourbonene	1388	1387	0.2			0.3
β-Elemene	1390	1389	2.5	0.2	0.1	2.2
7-*epi*-Sesquithujene	1392	1390		0.1		
Sesquithujene	1407	1405		0.1		
α-Gurjunene	1410	1409	1.5			
(*Z*)-α-Bergamotene	1412	1411		0.2		
β-Caryophyllene	1418	1417	8.9	2.8	0.9	2.3
β-Ylangene	1420	1419				5.0
β-Gurjunene	1432	1431	0.7	0.2	0.1	3.5
(*E*)-α-Bergamotene	1433	1432		0.4		
α-Guaiene	1439	1437	0.1			0.2
Aromadendrene	1440	1439	0.1		0.1	
(*Z)*-β-Farnesene	1442	1440		0.4		
6,9-Guaiadiene	1444	1442				1.2
(*E*)-Muurola-3,5-diene	1454	1451	5.9			
α-Humulene	1455	1452	2.0		0.4	1.3
Geranyl acetone	1456	1453			0.6	
(*E*)-β-Farnesene	1457	1454		4.7		
β-Santalene	1460	1457		0.1		
*allo*-Aromadendrene	1462	1458	0.9		0.2	
(*Z*)-Cadina-1(6),4-diene	1464	1461	0.1			0.6
Ishwarane	1467	1465				15.7
β-Acoradiene	1470	1469		0.2		
Dauca-5,8-diene	1473	1471		1.2		
(*E*)-Cadina-1(6),4-diene	1476	1475	1.6			
γ-Gurjunene	1477	1475				0.7
γ-Muurolene	1480	1478	0.3		0.1	1.0
Germacrene D	1486	1484	2.2		0.8	18.4
Aristolochene	1488	1487				2.1
β-Selinene	1490	1489	0.4		0.1	0.9
(*Z*)-β-Guaiene	1494	1492	3.8			1.4
α-Zingiberene	1496	1493		1.6		
Viridiflorene	1497	1496			0.3	
Bicyclogermacrene	1501	1500	1.2		0.5	5.1
α-Muurolene	1502	1500	0.5		0.2	1.7
β-Bisabolene	1506	1505		34.7	0.3	
(*Z*)-α-Bisabolene	1508	1506		0.4		
δ-Amorphene	1512	1511		0.3		
γ-Cadinene	1515	1513	0.2		0.1	0.8
Cubebol	1516	1514	0.1		0.1	0.5
7-*epi*-α-Selinene	1521	1520				7.5
β-Sesquiphellandrene	1523	1521		3.4	0.1	
(*E*)-Calamenene	1523	1521	6.1		0.1	0.3
δ-Cadinene	1524	1522	2.3		0.6	
(*E*)-*iso*-γ-Bisabolene	1530	1529		5.1		
(*E*)-γ-Bisabolene	1531	1530		35.0		
(*E*)-Cadina-1,4-diene	1534	1533	6.3			0.2
10-*epi*-Cubebol	1535	1533				0.1
α-Cadinene	1539	1537			0.1	0.2
α-Calacorene	1546	1544				0.3
Elemol	1549	1548	0.1			0.7
1-*nor*-Bourbonanone	1562	1561				0.1
(*E*)-Nerolidol	1563	1561			0.2	
β-Calacorene	1566	1564				0.2
Palustrol	1568	1567	0.3		0.3	0.2
Spathulenol	1578	1577	0.3	0.1	4.4	3.0
Caryophyllene oxide	1582	1582	0.1	0.2		0.8
Globulol	1591	1590	0.7	0.2		0.7
Viridiflorol	1592	1592	0.2	0.1	0.7	0.3
Cubeban-11-ol	1596	1595	0.2			
Rosifoliol	1601	1600		0.2	2.0	
Guaiol	1602	1600		0.8	0.1	
Ledol	1603	1602	5.0		0.3	0.3
Humulene epoxide II	1609	1608			0.2	0.6
Junenol	1620	1618				0.4
β-Cedrene epoxide	1621	1620				0.4
α-Corocalene	1623	1622				0.3
1-*epi*-Cubenol	1629	1627	0.7			0.5
α-Acorenol	1633	1632		0.1		
Gossonorol	1638	1636		0.3		
*epi*-α-Cadinol	1639	1638	0.6	0.3		0.8
Caryophylla-4(12),8(13)-dien-5α-ol						
and						
Caryophylla-4(12)-8(13)-dien-5β-ol	1640	1639			15.6	
*epi*-α-Murrolol	1643	1640	0.5	0.2		0.8
α-Muurolol	1645	1644	0.3			0.8
α-Cadinol	1654	1652	0.8	0.3	2.5	3.3
Selin-11-en-4-α-ol	1661	1658	0.2			0.8
*epi*-β-Bisabolol	1670	1670		0.4		
Bulnesol	1672	1670			0.7	
Cadalene	1676	1675				0.3
*epi*-α-Bisabolol	1684	1683		0.2		
Germacra-4(15),5,10(14)-trien-1-α-ol	1685	1685		0.1		0.6
α-Bisabolol	1686	1685		1.0		
Eudesma-4(15),7-dien-1-β-ol	1687	1687				0.3
2,3-dihydro-Farnesol	1688	1688			1.3	
*cis*-Thujopsenal	1708	1708		0.1		
(2*E*,6*Z*)-Farnesal	1714	1713		0.1		
5-hydroxy-(*Z*)-Calamenene	1715	1713	35.8			
(2*E*,6*Z*)-Farnesol	1716	1714			23.2	
(2*E*,6*E*)-Farnesal	1742	1740			1.8	
(2*E*,6*E*)-Farnesol	1744	1742			34.5	
(2*E*,6*E*)-Methyl farnesoate	1784	1783			0.7	
(2*Z*,6*E*)-Farnesyl acetate	1821	1821			0.3	
Monoterpene hydrocarbons						0.1
Oxygenated monoterpenes					0.2	
Sesquiterpenes hydrocarbons			53.3	91.6	6.3	77.9
Oxygenated sesquiterpenes			45.9	4.7	88.9	16.4
Total			99.2	96.3	95.4	94.4

RI_Calc._ = based on DB-5ms capillary column and alkane standards (C_8_-C_32_) according Van den Dool and Kratz (1963). RI_Lit._ = based on Adams (2007).

**Table 2:** Cytotoxic activity of the *Eugenia* oils on cell lines, after 72 h exposure.

*Eugenia* Species	*IC*_50_ (µg/mL) *				Hemolysis
	AGP-01	HCT-116	SKMEL19	MRC5	(µg/mL)
	(Gastric)	(Colon)	(Melanoma)	(Human Fibroblast)	
*E. egensis*	> 25	> 25	> 25	ND	> 200
*E. flavescens*	> 25	13.9 ^a^ (12.0–15.9)	> 25	14.0 ^a^ (10.4–18.6)	> 200
*E. patrisii*	> 25	16.4 ^b^ (14.6–18.3)	> 25	18.1 ^b^ (13.9–23.4)	> 200
*E. polystachya*	> 25	10.3 ^c^ (8.3–12.8)	> 25	>25	> 200
Doxorubicin	0.254 µM (0.19–0.33)	0.10 µM ^d^ (0.047–0.28)	0.045 µM (0.013–0.15)	0.20 µM (0.16–0.25)	>2 00 µM
		HCT-15/HT-29	Sbc-12/WM3211		
Eugenol		500.0 µM/300 µM	0.5 µM		

* Data are presented as *IC*_50_ values and 95% confidence intervals obtained by nonlinear regression for all cell lines, from three independent experiments. Doxorubicin and eugenol [41,42] were used as positive controls. Only compounds with *IC*_50_ values lower than 25 µg/mL, in at least one cell line, were considered active. ND = not determined. Values with different letters are statistically different at the *p* < 0.05 level (Tukey’s test).

## Abbreviations

The following abbreviations are used in this manuscript.

DMSO	dimethylsulfoxide
DPPH	2,2-diphenyl-1-picrylhydrazyl
GC-MS	gas chromatography/mass spectrometry
GC-FID	gas chromatography/flame ionization detector
MTT	3-(4,5-dimethylthiazol-2-yl)-2,5-diphenyl tetrazolium bromide
TEAC	trolox equivalent antioxidant capacity
Triton X-100	nonionic surfactant, octylphenol ethoxylate
